# TGF-β Signaling in Cancer: Control by Negative Regulators and Crosstalk with Proinflammatory and Fibrogenic Pathways

**DOI:** 10.3390/cancers11030384

**Published:** 2019-03-19

**Authors:** Hendrik Ungefroren

**Affiliations:** 1 First Department of Medicine, University Hospital Schleswig-Holstein, Campus Lübeck, D-23538 Lübeck, Germany; hendrik.ungefroren@uksh.de; Tel.: +49-451-3101-7866; 2 Clinic for General Surgery, Visceral, Thoracic, Transplantation and Pediatric Surgery, University Hospital Schleswig-Holstein, Campus Kiel, D-24105 Kiel, Germany

**Keywords:** TGF-β, signaling, EMT, phosphorylation, SUMOylation, fibrosis, cancer

## Abstract

The transforming growth factor-β (TGF-β) family of secreted growth factors controls many aspects of cell and tissue physiology in multicellular eukaryotes. Dysregulation of its pathway contributes to a broad variety of pathologies, including fibrosis and cancer. TGF-β acts as a powerful tumor suppressor in epithelial cells but during later stages of tumor development cancer cells eventually respond to this cytokine with epithelial-mesenchymal transition (EMT), invasion, metastasis, and immunosuppression. This collection of articles covers some important aspects of TGF-β signaling in cancer. Two articles focus on the role of TGF-β in tumor immunity and pro- and anti-inflammatory signaling, with one analyzing its impact on T-cell biology and different T-cell subsets, while the other deals with modulation of anti-inflammatory signaling by TGF-β receptors through proinflammatory signaling by immune receptors and the role of mechanotransduction in TGF-β-dependent immunosuppression. Another set of four chapters highlights the fact that context-dependent responsiveness to TGF-β is largely controlled by inputs from negative regulators and cooperation with proinflammatory and proapoptotic pathways. This theme is extended to the regulation of Smad signaling by differential phosphorylation, eventually converting canonical Smad signaling to a mitogenic, fibrogenic and carcinogenic outcome. Last, it is discussed how another posttranslational modification, SUMOylation, can modify protein function and impact TGF-β-induced EMT, invasion and metastasis.

Transforming growth factor-beta (TGF-β) is a pleiotropic cytokine that is produced in large amounts within cancer microenvironments. Its signaling pathway is among the key signal transduction pathways in cancer progression as exemplified by some tumor entities in which this pathway is altered in 100% of tumors [1]. In normal epithelial cells TGF-β acts as a tumor suppressor but during malignant conversion this role is switched to that of a tumor promoter due to mechanisms that are not well understood. Eventually TGF-β becomes a driver of neoplastic progression by enhancing tumor cell invasion, metastasis, cancer stem cell formation, genomic instability, and immune suppression. This phenomenon of a dual role in cancer has been termed the TGF-β paradox [2]. It is therefore not surprising that components of the TGF-β signaling cascade or factors that modulate their expression or activity were found to be key regulators of tumorigenesis. In fact, the targeting of the TGF-β pathway has come to the forefront as a bona fide therapeutic strategy. This is evident by the emergence of the TGF-β ligand and the TGF-β receptors as potential drug targets in a variety of malignancies, including metastatic colon cancer [3]. However, due its ubiquitous expression and trophic role in cell metabolism on the one hand and the tissue/cell type and tumor stage-specific functions of TGF-β on the other hand, a better understanding is mandatory for successfully targeting TGF-β signaling in cancer and at the same time avoiding serious side effects in patients.

In this Special Issue of *Cancers*, authors highlight major issues of TGF-β signaling in cancer: Two articles describe the role of TGF-β in tumor immunity and pro- and anti-inflammatory signaling. One focusses on various facets of T-cell biology and different T-cell subsets, while the other deals with this topic by looking at the interplay of anti-inflammatory signaling by TGF-β receptors with proinflammatory signaling by immune and death receptors. Another set of articles is devoted to positive and negative regulators of TGF-β signaling in prostate and pancreatic cancer. Finally, two chapters deal with TGF-β signaling modulation by posttranslational modifications, phosphorylation and SUMOylation.

TGF-β is well known for its ability to suppress the host’s T-cell immunosurveillance through inhibition of T-cell proliferation, activation, and their effector functions. Moreover, TGF-β also subverts T-cell immunity by favoring the differentiation of T-cell subsets, i.e., regulatory T-cells, that normally limit the antitumor response of cytotoxic T-cells. Intriguingly, recent studies provided evidence that TGF-β can also promote differentiation of certain inflammatory T-cell subsets, such as Th17, Th9, and resident-memory T-cells, which have been associated with improved tumor control in several models. Dahmani and Delisle [4] review recent advances in our understanding of the many roles of TGF-β in T-cell biology in the context of tumor immunity. Another prominent mode used by TGF-β for immunosuppression is inhibition of proinflammatory signaling and extracellular matrix (ECM) remodeling. Furler and coworkers [5] describe how activation of TGF-β activated kinase 1 (TAK1) lies at the crossroad of proinflammatory signaling by immune receptors and anti-inflammatory signaling by TGF-β receptors. Moreover, they discuss various concepts of mechanobiology of cancer. In addition to inhibiting proinflammatory signaling pathways within leukocytes, TGF-β can inhibit the immune system and support tumor growth through mechanical cues provided by the ECM to surrounding cells. Albeit ECM remodeling during cancer progression is crucial for tumor growth and metastasis, its extensive degradation can also promote inflammation. Understanding how TGF-β dampens proinflammatory responses and induces pro-survival mechanical signals throughout cancer development is critical for the development of therapeutics that block TGF-β or its signaling pathway with the intention to restore the host’s anticancer immune response and ultimately inhibit tumor progression.

TGF-β signaling is controlled at various levels and by both positive and negative inputs to enable cells to adapt activity of the pathway to physiologic stimuli and metabolic needs. Krüppel-like factor 10 (KLF10) represents an example for a positive mediator. It is a transcriptional regulator that binds to Sp1 sites on the DNA and interacts with other transcription factors. The review by Memon and Lee [6] describes the role and function of KLF10 in cancer with a special emphasis on its signaling with TGF-β. It is more and more appreciated that TGF-β signaling is subject to extensive, multi-level negative control and that aberrant regulation of inhibitory factors of the TGF-β1 signaling network (i.e., BAMBI, PPM1A, PTEN, SMAD7, SnoN) contributes to tumor progression. In their article, Tang et al. [7] discuss how these factors impact receptor function, Smad activation, intracellular signal bifurcation into canonical and non-canonical Smad, and non-Smad pathways and how they target gene promotor engagement. Authors also list the type of alterations such as loss of expression, overexpression, mislocalization, mutation or deletion of endogenous repressors that eventually result in modulation of signal intensity and/or duration. In addition, the authors discuss how aberrant regulation contributes to loss of growth inhibition and the epithelial phenotype, and to tumor progression/metastasis and speculate on the therapeutic potential of restoring the expression/function of these factors as a novel approach to cancer treatment. Kardooni and colleagues [8] provide an example for inhibition of TGF-β/Smad-dependent transcriptional activity by reinduction of Dickkopf-related protein 3 (DKK3) expression through two approaches, promoter demethylation by treatment of PC3 prostate cancer cells with a DNA methyltransferase (DNMT) inhibitor and via direct induction using CRISPR and Cas9-VPR. Cas9-VPR is a modified version of Cas9 that can induce expression of the guide RNA target gene rather than edit its genomic sequence. Interestingly, in both cases, re-expression of DKK3 resulted in attenuation of PC3 cell migration and proliferation. This example shows that TGF-β by increasing DNMT expression can reactivate genes that are downregulated by promoter methylation. From a methodological perspective it is noteworthy that CRISPR/Cas9-VPR-mediated induction of DKK3 represents a potential therapeutic tool for prostate cancer. Radke and coworkers [9] employing a pancreatic carcinoma cell model provide another paradigm for negative regulation of TGF-β signaling. They observed that knockdown of TNF receptor apoptosis-inducing ligand receptor 1 (TRAIL-R1) in pancreatic cancer cells led to an increased abundance of the TGF-β type II receptor (TβRII) and was associated with enhanced TGF-β1-induced expression of plasminogen activator-inhibitor 1 and growth inhibition. Interestingly, the death receptor TRAIL-R1 suppressed TβRII through induction of microRNA-370-3p, which in turn decreased TβRII expression. Notably, TRAIL-R1 besides its well-known ability to induce cell death following binding of its ligand TRAIL can also trigger a variety of cell death-independent cellular responses such as activation of proinflammatory gene transcription [10]. Notably, TAK1 may represent a switch that allows the cell to activate proinflammatory RelA/p65, JNK, and p38 MAPK signaling in response to TRAIL stimulation [11] while blocking TRAIL-induced cell death [11,12]. Thus, TRAIL-R1-induced downregulation of TβRII provides another (reverse) variation of the theme of crosstalk of proinflammatory death receptor with anti-inflammatory TGF-β signaling. Inhibiting TRAIL-R1 expression or function could thus represent a potential strategy to either enhance TGF-β’s tumor suppressor function or block hyperactive and detrimental TGF-β signaling depending on tumor type and stage of malignancy (see TGF-β paradox). 

Rather than altering TGF-β signaling at the level of individual components of the network, functional activities may also be altered as a result of posttranslational modifications such as phosphorylation and SUMOylation. A prominent example is represented by TGF-β/Smad signaling in chronic liver injuries. These can result from viral infection which induces TGF-β to promote liver fibrosis and eventually hepatocellular carcinoma (HCC). Yoshida and colleagues [13] report that these insults are associated with differential phosphorylation of receptor-regulated Smad proteins (R-Smads) resulting in three types of phosphorylated R-Smads: C-terminally phosphorylated Smad2/3 (pSmad2C and pSmad3C), linker phosphorylated Smad2/3 (pSmad2L and pSmad3L), and dually phosphorylated Smad2/3 (pSmad2L/C and pSmad3L/C). These various phospho-Smad isoforms seem to possess different functional activities. For instance, TGF-β-induced pSmad2/3C signaling has cytostatic function, while cytokine-induced pSmad3L signaling accelerates cell proliferation and promotes fibrogenesis. Remarkably, pSmad3L signaling resulting from viral infection may be reversed following antiviral therapy and could thus represent a biomarker of tissue regeneration/healing. Finally, Chanda and coworkers [14] devote their attention to protein posttranslational modification by the small ubiquitin-like modifier (SUMO) and discuss studies showing that SUMOylation can regulate the stability, subcellular localization or interactome of a protein substrate with key consequences for cellular processes including TGF-β-induced epithelial-mesenchymal transition (EMT). Interestingly, SUMOylation of TβRI/ALK5 was suggested to be important for TGF-β-induced invasion and lung metastasis of Ras-transformed fibroblasts. Further, SUMOylation of SnoN, a Smad-interacting protein that negatively regulates TGF-β signaling [7], appears to be crucial for suppression of TGF-β-induced EMT and invasive growth in breast cancer cells. The authors conclude that the intersection of TGF-β-induced EMT and the SUMO system has important implications for cancer formation and progression, and possibly the identification of novel therapeutics.

In summary, this Special Issue of Cancers is a collection of articles discussing various aspects of TGF-β signaling in cancer biology. A better understanding of the complexity of TGF-β signal transduction, particularly its context-dependency and multi-level control, is mandatory to be able to successfully interfere with only its oncogenic, “evil” pathways and regulators. Only then approaches to target TGF-β for antiangiogenesis, antimetastasis and restoration of anticancer immunity will have a chance to benefit patients.
